# Controlled transitions between metastable states of 2D magnetocapillary crystals

**DOI:** 10.1038/s41598-022-20035-8

**Published:** 2022-09-26

**Authors:** Ylona Collard, Franco N. Piñan Basualdo, Aude Bolopion, Michaël Gauthier, Pierre Lambert, Nicolas Vandewalle

**Affiliations:** 1 GRASP, Institute of Physics B5a, Université de Liège, 4000 Liège, Belgium; 2 TIPs, École Polytechnique de Bruxelles, Université Libre de Bruxelle, 1050 Brussels, Belgium; 3 FEMTO-ST, CNRS, Université Bourgogne Franche-Comté, 25000 Besançon, France

**Keywords:** Applied physics, Condensed-matter physics, Statistical physics, thermodynamics and nonlinear dynamics, Condensed-matter physics, Soft materials, Structural materials

## Abstract

Magnetocapillary interactions between particles allow to self-assemble floating crystals along liquid interfaces. For a fixed number of particles, different states possessing different symmetrical features, known as metastable states, coexist. In this paper, we demonstrate how to trigger the transition from one state to another, either by rearranging the crystal, or by controlling its growth. First, we show that externally controlled magnetic fields can squeeze the entire crystal to induce structural modifications, that upon relaxation can lead to a modified state. Second, we propose localized laser-induced thermocapillary flows that can be used to guide new particles towards an existing crystal in a desired direction, thus favoring a particular resulting state. The control of the formation of metastable states is a key ingredient to functionalize such assemblies, paving the way to self-assembled microrobots.

## Introduction

Multiple particles systems are of interest in several areas of physics. Their structure is governed by pairwise interactions. In particular, in the presence of attractive interactions, these systems tend to self-assemble, minimizing their energy. This phenomenon exists at all scales, governing the formation of molecules and planetary systems^1^. Depending on the complexity of the interactions, particles may form simple periodical structures (crystals), or more complex ones as protein chains^2^.

Self-assembly has caught the interest of academia and industries because of its use to fabricate tiny structures. Indeed, some structures are too large to be prepared by chemical synthesis while too small to be assembled by robotic methods. In particular, the micrometer–millimeter scale is typically the bottleneck between bottom-up and top-down standard fabrication methods^3,4^. One of the main characteristics of self-assembling systems is that due to the high number of degrees of freedom, on top of the global minimal energy state, there often exist several local minima. These metastable states can be observed at all scales, at the molecular level^5,6^, in colloids^7^, at mesoscale and at macroscopic scales^8^. Interest on how to exploit these metastable states for active structuring^9^ has recently been growing. Therefore, a fundamental question, that we address in this paper, is to define the conditions enabling to navigate between the different metastable states.

**Figure 1:**
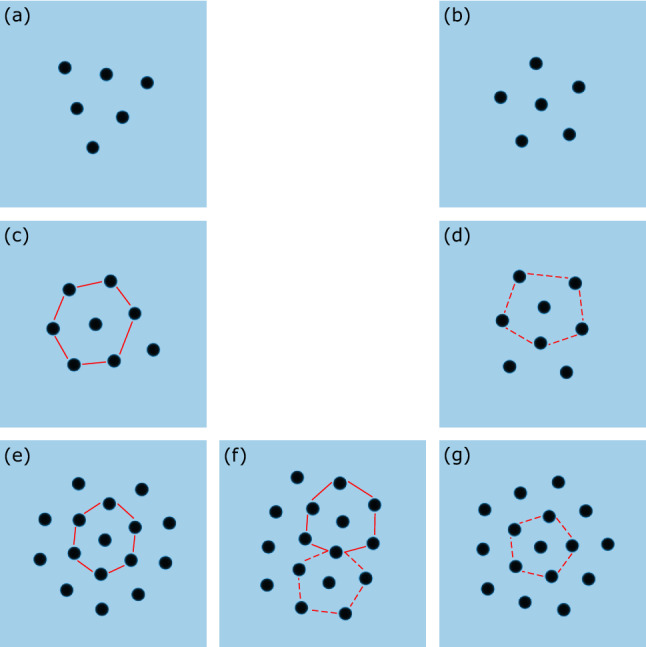
Experimental pictures of magnetocapillary self-assemblies’ typical equilibrium states. These assemblies are made of a finite number *N* of particles. (**a**,**b**) Two coexisting states for $N=6$. (**c**,**d**) Two coexisting states for $N=8$. (**e**–**g**) Three coexisting states for $N=16$. Pictures on the left column (**a**,**c**,**e**) show that central particles tend to form a triangular lattice, while pictures of the right column (**b**,**d**,**g**) show central particles having a five-fold symmetry. Picture (**f**) shows a combination of beads with 5 or 6 neighbors in the center of the assembly.

In this paper, we consider magnetocapillary driven self-assemblies of mesoscale particles at air–water interface. It is inspired by the pioneering experiment of Alfred Marshall Mayer published in 1878^10–12^ often cited by Sir Thomson and Lord Kelvin in their respective models on the atomic structure^13–18^. In the present work, the particles are 400 or 500 $\upmu \mathrm {m}$ diameter soft ferromagnetic beads floating at a horizontal air–water interface under a vertical constant magnetic field $B_z$. Each particle is attracting the others by capillary interactions, also known as Cheerios effect^19,20^. The imposed vertical constant magnetic field induces parallel dipoles inside the particles, providing a repulsive magnetic interaction between them. Although the use of non-homogeneous magnetic fields can be of interest for micromanipulation purposes^21^, in this work we focus on the homogeneous magnetic field case (i.e., no confinement potential). The interaction between two particles *i* and *j* at distance $r_{ij}$ can be described by a dimensionless potential^22–24^:

1 $$\begin{aligned} u_{ij} = -K_0\left( x_{ij} \right) + \frac{{\mathrm{Mc}}}{x_{ij}^3}\,, \end{aligned}$$

where $x_{ij}={r_{ij}/\lambda }$ is the normalized distance between the beads in the horizontal plane, in capillary length units $\lambda =\sqrt{\gamma /\rho g}$. The first term $K_0$ of the right-hand side of Eq. (1) accounts for the capillary attraction, and the second, for the repulsive dipole-dipole magnetic interaction. The parameter ${\mathrm{Mc}}$ in Eq. (1) is the magnetocapillary number, capturing the competition between magnetic and capillary effects. This number can be tuned by the vertical field since $\mathrm Mc = \kappa B_z^2$, where the coefficient $\kappa = 0.0027 \, \mathrm{mT^{-2}}$, independent of the bead size, has been calibrated in an earlier work^24^. The balance between the capillary and magnetic interaction induces long-range attraction and short-range repulsion—similar to molecular interactions—leading to the formation of 2D floating crystals.

In this work, we study the stable and metastable states of crystals with a small number of particles *N* (from 3 to 19). For certain numbers *N*, the particles are observed to stabilize in different metastable states depending on the initial conditions, some of them being less frequent than others. Figure 1 shows typical floating crystals obtained for a finite number of particles *N* and starting from different initial conditions. This collection of pictures emphasize that different metastable states coexist for a finite *N*. The most striking observation is that the central particles can be surrounded either by 6 neighbors (left column), by 5 neighbors (right column) or by both 5 or 6 neighbors (f). Although variations on the arrangement of the central particles have been observed in the case of confined repelling objects^25^, in our unconfined case, these variations can affect the shape of the entire floating crystal. The final state has been noted to be extremely sensitive to the initial distribution of particles, with some states rarely obtained from random initial conditions. Therefore, a controlled way of transitioning between states could be useful to study these metastable structures.

**Figure 2:**
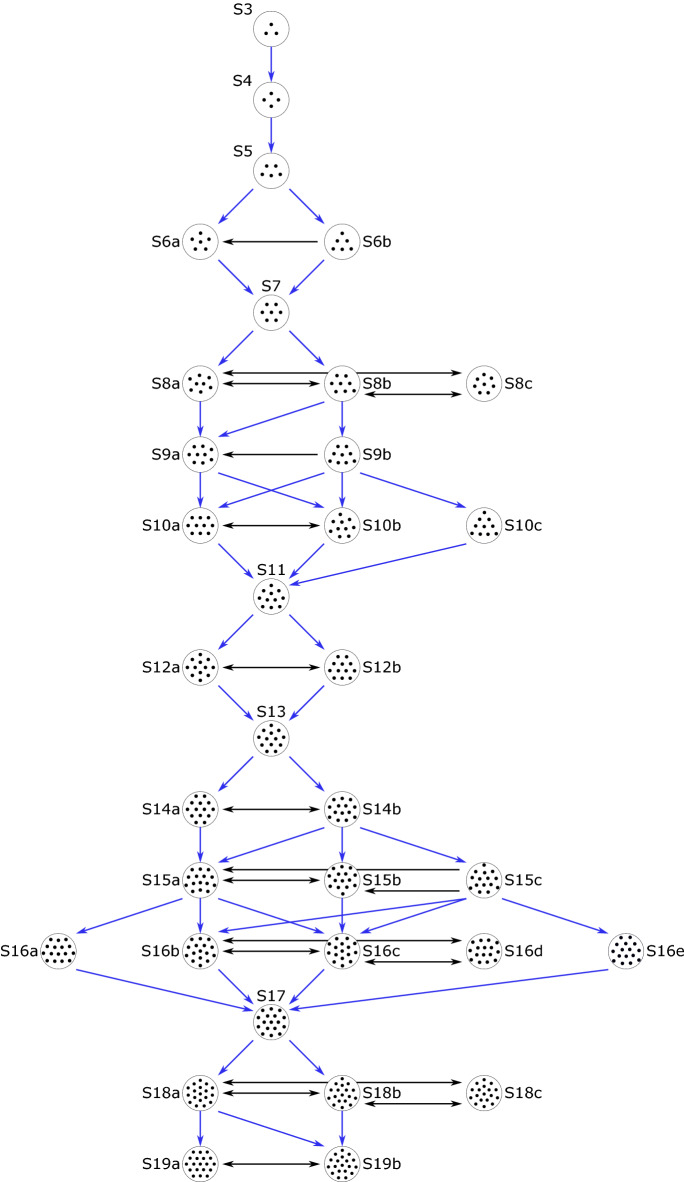
The various structures adopted by a floating crystal for a number of particles between $N=3$ to $N=19$. Each state is named as the letter “S” followed by a number, which indicates the number of particles, and, if necessary, a letter to distinguish different states with the same number of particles. The possible transfers between states having the same number *N* of particles (black arrows) are obtained with the magnetic reconfiguration method. Double black arrows indicate that it is possible to switch from one state to another and vice versa. Single black arrows give the only obtained direction of transition between the two states. The possible transfers from states having *N* particles to ones having $N+1$ particles (blue arrows) are obtained with the controlled growth method.

The contribution of this work is to control the formation of such floating crystals, and the transitions between metastable states. Two complementary approaches are demonstrated: (i) for a given number of particles, magnetic actuation can trigger transitions between metastable states, and (ii) laser-induced thermocapillary flows can be used to control the growth of floating crystals. All the experimentally observed transitions are summarized in Fig. 2. Each state is named as the letter “S” followed by a number, which indicates the number of particles, and, if necessary, a letter to distinguish different states with the same number of particles.

## Results

### Magnetic reconfiguration

**Figure 3:**
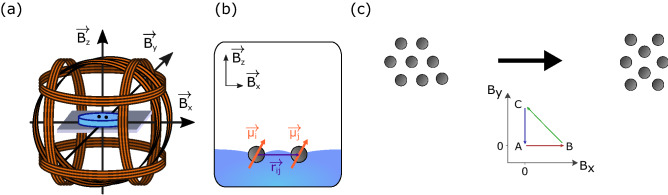
(**a**) First experimental setup composed of three Helmholtz coils producing uniform magnetic fields $(B_x,B_y,B_z)$ in the center of the system where the floating crystal self-assemble. (**b**) Influence of a horizontal magnetic field on the magnetic interaction between two particles. (**c**) The cycle ABC in the $(B_x,B_y)$ plane induces structural modifications in the floating crystal and a probability of changing state.

To study transitions between metastable states, a first setup consists of a tri-axial Helmoltz coil system, as illustrated in Fig. 3a, to impose an arbitrary external magnetic field and adjust the magnetocapillary pairwise interaction. Indeed, adding a $B_x$ or a $B_y$ component will modify the potential *u* resulting in a global deformation of the floating structure. For example, when some horizontal field component $B_x = \beta B_z$ is switched on, as illustrated in Fig. 3b, the interaction potential between two particles from Eq. (1) becomes

2 $$\begin{aligned} u_{ij}= -K_0(x_{ij}) + \frac{\mathrm{Mc}}{ x_{ij}^3} \left( 1+\beta ^2-3\beta ^2 \cos ^2 \theta _{ij} \right) \end{aligned}$$

where $\theta _{ij}$ is the angle formed by the x-axis and the vector $\mathbf {r}_{ij}$ linking the dipoles. One should remark that the magnetic interaction may become attractive for $\beta >1/\sqrt{2}$, a case which is not considered herein to avoid the irreversible collapse of particles.

A global deformation of the crystal can be achieved by following a cycle in the horizontal field plane $(B_x, B_y)$ as shown in Fig. 3c. After that manipulation, there is a certain probability that the crystal switches to a new metastable state. To trigger a deformation and reorganization of the structure, the amplitude of $B_x$ and $B_y$ fields must be large enough. Therefore, there is a threshold amplitude, depending on *N* and $B_z$, which allows the assembly to change state. Typical field values are given in Supplementary Materials section A.

**Figure 4:**
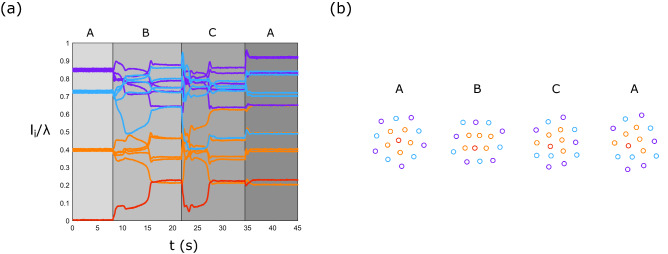
Experimental results about the transition between S16b and S16d for $B_z=5\,\mathrm{mT}$. (**a**) Measured normalized distance ($l_i/\lambda$) of each bead *i* to the center of mass of the assembly as a function of time. Four steps will be observed corresponding to stages A, B or C of the cycle in the $(B_x,B_y)$ space (see Fig. 3c). These phases are shown on the graph and corresponding assembly stable states are drawn in (**b**). The beads and their normalized distance ($l_i/\lambda$) are colored according to their distance from the center of mass in the initial state S16b.

The magnetic reconfiguration technique to transit between metastable states is illustrated in Fig. 4, using assemblies of $N=16$ beads. To visualize this transition, the distance $l_i$ of each bead *i* to the center of mass of the whole crystal has been measured, providing a set $\{\frac{l_i}{\lambda }\}$ containing the 16 beads relative distance $l_i$ divided by the capillary length $\lambda$. Every metastable state is characterized by a defined set $\{\frac{l_i}{\lambda }\}$. The graph of the set $\{\frac{l_i}{\lambda }\}$ as a function of time is given in Fig. 4a for the transition between S16b and S16d. Four steps were observed in the transition corresponding to stages A, B or C of the cycle in the $(B_x,B_y)$ space (see Fig. 3c). In Fig. 4b are drawn the stable states of the assembly corresponding to the stages A, B, C and A. The beads and their normalized distance ($l_i/\lambda$) are colored according to their distance from the center of mass in the initial state S16b. Given the symmetry of the assembly, the central bead is located at the center of mass of the structure. It will be colored in red. The five first neighbors of the center of mass will be colored in orange, the five second in blue and the last five in purple. These colors allow us to follow the relative position of the particles and their situation in the assembly over time. During phase (A) the assembly is in its initial state S16b, there is no horizontal magnetic field deforming the structure. Then, a horizontal magnetic field $B_x$ is applied and during this phase (B) the assembly deforms and rearranges itself. The beads become closer to each other under the effect of the horizontal magnetic field. After a certain time (around 8 s) the structure becomes stable (the beads stop moving) but is still deformed by the field. In phase (C), the orientation of the horizontal magnetic field is rotated by 90$^{\circ }$. The assembly also changes its orientation, and a reorganization is observed. The return to phase (A) can be achieved by the extinction of the horizontal magnetic field $B_y$. The beads move away from each other and towards their final position, the state S16d. The experiment enables concretely to transition from state S16b to state S16d. We can notice that the set $\{\frac{l_i}{\lambda }\}$ of S16b and S16d are completely different.

Using the magnetic reconfiguration technique, we were able to create the configurations tree shown in Fig. 2. This method enables to navigate horizontally between different states on this tree and the possible transitions are described by the black horizontal arrows. A double black arrow means that it is possible to switch with this experimental protocol from one state to the other and vice versa. A single black arrows gives the only obtained direction of transition between the two states. This indicates that certain structures (S6b, S9b and S15c) are only obtained when the beads are deposited on the water surface and that the technique of magnetic reconfiguration of the assembly does not achieve these states.

Numerical simulations were performed to confirm the experimental results and to allow a more in-depth study of the influence of the parameters. The simulations methods and results are presented in section C of Supplementary Materials. It can be seen that the numerical results confirm quite well our experimental observations.

### Controlled growth

The second method consists of using laser-induced thermocapillary actuation^26^ to control a single free particle (i.e., far away from other particles) and guide it towards an existing floating crystal. By adding the free particle to the crystal in an appropriate direction, we can favor the emergence of one state or another.

**Figure 5:**
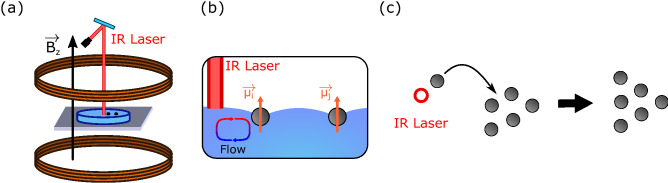
Controlled growth setup. (**a**) Second experimental setup composed of one pair of Helmholtz coils to produce a vertical magnetic field $B_z$ and an IR laser to create a hot spot on the liquid interface. (**b**) Side view of the use of the laser-induced thermocapillary flow to bring a magnetic particle closer to another one. The flow pushes the particle away from the laser spot. (**c**) Developed strategy to achieve a desired final state by adding a particle to an existing crystal in a particular direction.

The experimental setup, presented in Fig. 5, consists of a water container surrounded by one pair of Helmholtz coils to generate a vertical magnetic field. Additionally, an infrared (IR) laser is pointed towards the recipient from above, enabling to locally heat the air–water interface. The energy of the laser is absorbed by the water, locally increasing the interface temperature, and thus generating an interfacial thermocapillary flow^27^, as schematized in Fig. 5b. This flow would propel floating particles away from the laser spot and can be used to control the trajectory of floating particles by displacing the laser spot with a piezoelectric tip/tilt mirror^26^. Namely, this system can guide free particles towards a floating crystal in defined directions to favor a particular resulting state, controlling its growth.

**Figure 6:**
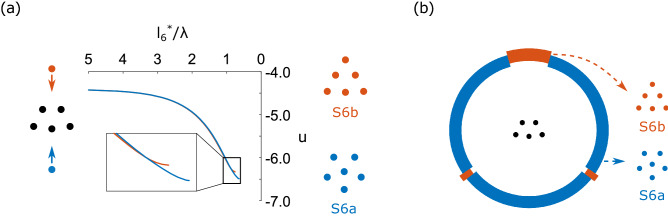
Dynamic simulation results for the transitions from S5 to S6a and S6b for an external magnetic field $B_z=5\,\mathrm{mT}$. The sixth particle is placed at a distance of $5\lambda$ from the initial assembly and, depending on the direction at which the new particle is added, the system will stabilize in one state or the other. (**a**) Dimensionless potential *u* as a function of the normalized distance of the sixth particle to the center of mass of the five other particles $l^*_6/\lambda$. Two different cases are shown: additional particle up and down, leading to the formation of two different states S6a and S6b. (**b**) Predicted final state as a function of the angle at which the sixth particle is added.

To illustrate the controlled growth principle, the transition between S5 and S6a or S6b is considered. First, dynamic numerical simulations (presented in details in Supplementary Material section C) were performed to predict the evolution of the system upon the addition of a new particle. In Fig. 6a two basic scenarios are analyzed: adding a particle from one side or the other of an existing S5 crystal, obtaining a S6a crystal in one case and or S6b crystal in the other. The transition and final state are analyzed using the total dimensionless potential *u* (Eq. 1) as a function of the normalized distance $l^*_6$ between the new particle and the center of mass of the five original ones. Both the distance $l^*_6$ and the potential *u* decrease until reaching their equilibrium value, which is different for each final state. It can be seen that the two final states differentiate only at the end of the trajectory, close to equilibrium. This can be explained by the fact that the energy excess of a free particle is much larger than the energy difference between metastable states. Then, we analyzed numerically the reached metastable state as a function of the direction at which the new particle is added. The results are shown in Fig. 6b, where it can be seen that the transition to the state S6a is much more common. This result, however, highlights the potential of this controlled growth technique to obtain different metastable states by controlling only the direction from which the free particle approaches the original assembly.

**Figure 7:**
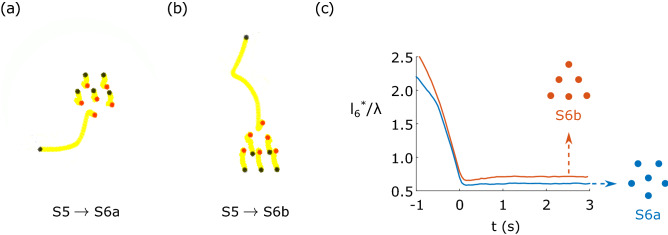
Experimental results showing the transitions from S5 to S6a and S6b for $B_z=4.8\,\mathrm{mT}$. In black, the initial particles positions, in yellow the particles trajectories and in red the final state. In both cases, the sixth particle was approached to the existing S5 crystal in the desired direction using the thermocapillary actuation system. (**a**) Transition from a S5 to S6a. (**b**) Transition from S5 to S6b. (**c**) Normalized distance $l^*_6/\lambda$ between the new particle and the center of mass of the other five particles, in function of time.

Experimental results of the previous example, i.e. the transition from S5 to S6a or S6b, are shown in Fig. 7. We replicated the numerical results by guiding a particle towards an existing a S5 crystal in the appropriate directions to favor the transitions to S6a and S6b, as shown in Fig. 7a,b, respectively. The evolution of $l^*_6/\lambda$ with time is shown in Fig. 7c, where it can be seen that both cases evolve similarly in the approaching phase, but they stabilize at different values. As previously mentioned, the energy excess of a free particle is always much higher than the energy difference between states. In simulations, we neglected the inertia of the particles (quasi-static process). Experimentally, however, the kinetic energy of the approaching particle can perturb the assembly process, which can result in a state other than the expected one. This is especially noticeable when trying to obtain the less stable states, like S6b. Therefore, although in simulations the controlled growth strategy allowed us to choose the resulting state in a deterministic way, experimentally we may need several trials before reaching the less stable states.

With this method, we studied experimentally the possible transitions from a crystal with *N* particles to one with $N+1$ particles with *N* ranging from 3 to 18, completing the configurations tree shown in Fig. 2. The controlled growth method allowed us to navigate the three vertically, in the increasing size direction, as shown by the blue arrows. A video compiling all the transitions is proposed in the Supplementary Materials. Although with this method, some states were not observed (S8c, S16d and S18c), they differ from the ones that were not reachable with the first technique. Therefore, we conclude that both techniques complement each other, allowing us to navigate the entirety of the configurations tree. The dynamic simulation results for *N* from 3 to 18 are described in the Supplementary Material section C. It can be seen that, besides a few exceptions, the numerical results confirm our experimental observations.

## Discussion

In this work, we have studied the different metastable states of 2D self-assembled crystals of particles, paying particular attention to the transitions between states. We have proposed two different and complementary approaches to control transitions between different metastable states. The first one is the magnetic reconfiguration method. By adding a horizontal magnetic field, we can break the isotropy of pairwise interactions, compressing the assembly in the direction of the horizontal field. Upon relaxation, the assembly can transition to a different state. Second, by using laser-induced thermocapillary flows, we can add new particles to an existing assembly in a controlled manner to favor some resulting states over others, controlling the growth of the crystal.

In the first method, the switch between states is still a probabilistic process and it appears difficult to accurately predict the outcome of an experiment. Sometimes the method needs to be applied few times to obtain the wanted final state. Similarly, some transitions are not reachable using the controlled growth technique. However, combining both tools (magnetic triggered reconfiguration and controlled growth) enables to reach all the observed metastable states for crystals from 3 to 19 particles and also to navigate between almost all the states. As an example, reaching S9b from S8a could be achieved by first transitioning from S8a to S8b using the magnetic reconfiguration principle, and then transfer to S9b using the controlled growth technique. The transfer between metastable states considering a decremental number of beads is also possible. Indeed, the laser-induced thermocapillary actuation can also be used to remove a particle from an assembly. However, the disturbances induced by the flow make the resulting $N-1$ state unpredictable. For example, removing a particle from a S9a could randomly render a S8a or S8b. Nevertheless, the magnetic-based reconfiguration could be used afterward to choose the desired metastable state.

Since the number of metastable states grows rapidly with *N*, listing all the possible states of larger crystals becomes complex. Large assemblies are normally described as a perfect hexagonal crystal with defects or, alternatively, as one of the smaller assemblies surrounded by several layers of beads. Therefore, in this study, we focused on few particles crystals (up to 19), since in these cases, the different states present different shapes and symmetries and can be easily differentiated. Moreover, it has been proven that on crystal nucleation and growth, the organization of the first few molecules can have an impact on the geometry of the final crystal^28^.

The study of crystals’ metastable states and how to transition between them can be of interest to many areas of science. The similarity between the magneto-capillary potential (Eq. 1) and the molecular interaction potentials make this system a promising accessible physical model to study crystalline solids. Also, we showed in previous works that our system can be downscaled and that the lower limit is a bead diameter of few micrometers. In that case, our system could be considered as a colloid crystal. The assembly of colloidal particles into various types of lattices/arrays is one of the fundamental challenges of colloid science given the potential applications of such structures in phononics^29,30^, photonics^31–33^, electronics^34^ and sensors^35,36^. A lot of strategies have been developed to create colloidal lattices and assemblies^37–45^. Although it is possible to position colloids into desired structures using assembly schemes^46–48^, reversible dynamic control of the position, symmetry and structure of the assembly remains challenging^49^. Our study, proposing two different techniques for reversible state change, could therefore have an impact in this field.

Finally, it has been showed that these floating crystals are able to perform tasks such as motion, cargo transport and fluid mixing^24,50,51^. The shape, size and symmetry are key ingredients to functionalize such assemblies. More particularly, at low Reynolds number, the structure should oscillate between different states to achieve a motion, that is, to swim. By showing that different states exist for different *N* values and by demonstrating how to switch from one structure to another one, we provide ways to animate/functionalize floating crystals. Moreover, the two techniques presented in this article (magnetic reconfiguration and controlled growth) leave many possibilities open. They could be used to manipulate non-spherical particles and mixtures of particles of different sizes and shapes.

## Supplementary Information

Supplementary Information 1.

Supplementary Information 2.

## Data Availability

The datasets generated during and/or analysed during the current study are available from the corresponding authors on reasonable request.
