# Supplementary material for: Controlled transitions between metastable states of 2D magnetocapillary crystals
Source: Sci Rep. 2022 Sep 26;12:16027. doi: 10.1038/s41598-022-20035-8 (PMC9513081; doi:10.1038/s41598-022-20035-8)
Supplement: Supplementary file 1 — Supplementary Information 1. [file 41598_2022_20035_MOESM1_ESM.pdf]

Controlled transitions between metastable states of 2D magnetocapillary crystals

Ylona Collard[1,+,*], Franco N. Piñan Basualdo[2,3,+,*], Aude Bolopion[3], Michaël Gauthier[3], Pierre Lambert[2], and Nicolas Vandewalle[1]

[1]GRASP, Institute of Physics B5a, Université de Liège, B4000 Liège, Belgium.
[2]TIPs, École Polytechnique de Bruxelles, Université Libre de Bruxelle, 1050 Brussels, Belgium.
[3]FEMTO-ST, CNRS, Université Bourgogne Franche-Comté, F-25000 Besançon, France.
[*]ycollard@uliege.be, Franco.Pinan.Basualdo@ulb.be
[+]these authors contributed equally to this work

Supplementary materials

A. Magnetocapillary setup

The first experimental setup, enabling to switch from one metastable state to another, is the following. A glass container filled with water is placed in the center of a large triaxial Helmholtz coils system, as illustrated in Fig. 3a. A picture of the experimental setup is shown in Fig. S1a. By injecting current into the coils, spatially uniform magnetic fields can be generated inside the system in any direction. We place soft ferromagnetic spheres (of 400 or 500 μm diameter) at the air water interface, where they float thanks to surface tension[1] and attract each other because of capillary interactions[2]. Magnetic dipoles of controllable magnitude and direction can be reversibly induced in the particles through the external magnetic field[3]. The z-coil is used to generate a constant and vertical magnetic field B_z in order to create a magnetic repulsion and to avoid the contact between the particles[4–8].

The first experimental method is explained in Fig. 3b and 3c. A magnetocapillary assembly of N particles is created on a water surface inside the experimental setup. Initially, the magnetic field inside the setup points only in the z-direction. These conditions correspond to point A in the diagram of Fig. 3c. Then, a horizontal magnetic field B_x is applied along the x-axis by injecting current in x-coils. This action leads the system to the point B in the diagram. The addition of a horizontal magnetic field is shown in Fig. 3b. This provides extra interactions along the horizontal plane such that the structure is squeezed in the x direction. After that, the in-plane field is turned by 90 degrees by injecting current in the y-coils instead of the x-coils. It creates a magnetic field B_y leading the system to point C and squeezing the assembly in the y direction. The last step is to shut down the in-plane magnetic field and go back to point A. After this cycle the structure had the opportunity to jump from a metastable state to another as it is presented in Fig. 3 and 4. To trigger a deformation and reorganization of the structure, the amplitude of the B_x and B_y fields must be large enough. Therefore, there is a threshold amplitude, depending on N and B_z, which allows the assembly to change state. Typical field values are $B_x, B_y \in [0.3\text{mT}, 3\text{mT}]$ for $B_z \in [3\text{mT}, 10\text{mT}]$.

B. Thermocapillary setup

The second technique used to navigate between metastable states is the controlled growth by thermocapillary actuation. The actuation principle is illustrated in Fig. 5 and a picture of the experimental setup is shown in Fig. S1b. A container filled with water is placed in the center of one pair of Helmholtz coils orientated vertically. Also, a robotized infrared laser beam (wavelength $\lambda = 1455\,\text{nm}$, waist diameter $d_L = 1.3\,\text{mm}$) is pointed towards the water surface enabling to locally heat the air-water interface and triggering a thermocapillary flow[9,10]. We place soft ferromagnetic spheres (of 500 μm diameter) at the air water interface, where they float because of surface tension[1] and attract each other because of capillary interactions[2]. The generated vertical magnetic field B_z induces a vertical magnetization of the spheres, creating a repulsive magnetic interaction among the particles and preventing them from contact[4–8]. Finally, the flow generated by the laser tends to push floating particles away from the laser spot. Indeed, by displacing the laser spot we can achieve the precise control of a particle's trajectory[11], which can be used

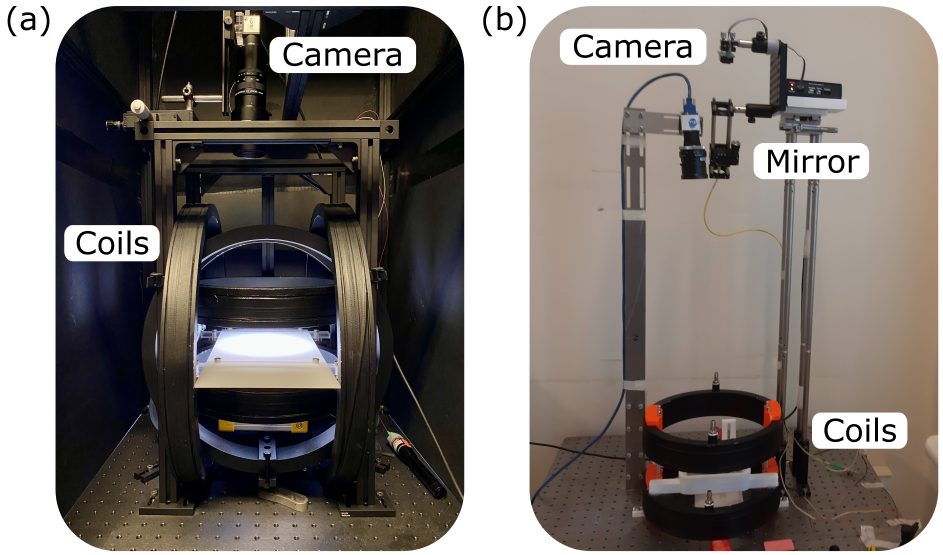

Figure S1. Picture of the magnetocapillary setup (a) and the thermocapillary setup (b).

to explore the metastable states.

The methodology consists in several steps. First, a given number of particles is placed at the air-water interface with the magnetic field B_z on. Then, they are left to assemble into one of the possible states. Finally, a new particle is guided by the laser spot towards the existing crystal. By controlling the direction at which the new particle is added to the crystal, we can favor one or other resulting state, controlling the growth of the crystal.

Notice that, contrary to the magnetocapillary interactions, the thermocapillary actuation is based on gradients of surface tension rather than on its absolute value. Considering the used laser powers ($P_L < 150\,\mathrm{mW}$), the measured temperature increase is $\Delta T < 6\,\mathrm{K}$, and the resulting surface tension variation is $\Delta\gamma < 1\,\mathrm{mN/m}$[10]. Therefore, we can neglect the variation in surface tension due to the laser heating and assume the magnetocapillary interactions to remain unchanged. Although the laser could deform the interface[12], since the change in surface tension is small ($< 1.5\,\%$), this deformation can be neglected.

Both techniques (in-plane magnetic field or robotized thermocapillary actuation) are complementary as the first enables a reconfiguration of the crystal, inducing state transition, and the second is able to precisely guide a new particle to a crystal, controlling its growth.

C. Numerical model

In order to validate our experimental results and analyze the different possible states of an assembly, models can be implemented to calculate the local or total energy of the structure.

The models used in this work are based on three main assumptions : (i) hydrodynamic effects are neglected since only equilibrium states are searched for, (ii) the superposition principle is considered to calculate the total magnetocapillary potential since particles are well separated, and (iii) identical particles are roughly at the same elevation in water.

The magnetocapillary interaction between two identical soft-ferromagnetic particles, floating at some liquid-air interface under a constant vertical field B_z, is described in Eq. 1. When some horizontal field component $B_x = \beta B_z$ is switched on, the interaction potential changes and is described in Eq. 2. Both expressions are used to build static and dynamic model describing the transition between two states. Static simulations are used to model the magnetic reconfiguration method while dynamic simulations are used to describe the controlled growth method.

Static modeling

Taking the dimensionless potential into account, we start simulations with random dispersion of particles. Thereafter, simulations consider a steepest descent algorithm to minimize the magnetocapillary potential. At each simulation

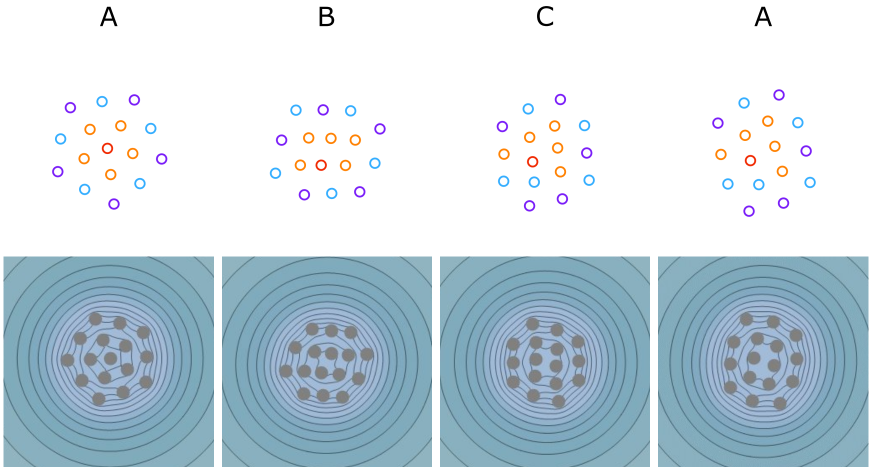

Figure S2. Stable states of an assembly during the different steps of the S16b-S16d transition for an external magnetic field $B_z = 5\,\mathrm{mT}$. Four steps will be observed in the transition corresponding to stages A, B, C and A of the cycle in the (B_x, B_y) space (see Fig. 3c). The first line of configurations correspond to experimental results and the second line correspond to simulations. In the experimental results, we have colored the beads according to their distance from the center of mass in the initial state S16b.

step, the total potential u is computed:

$$u = \sum_{\forall i,j;i \neq j} u_{ij}, \tag{S1}$$

where u_{ij} is the dimensionless interaction potential between two beads (described in Eq. 2). Then the x, y positions of the particles are slightly randomly modified and the new value of potential u is calculated. If the new potential is below its previous value, then the temporary positions are recorded, otherwise the system returns to the previous positions. After several iterations, the system finds a minimum for u. This minimum could be a local or global minimum as obtained in experiments.

This technique can be used to model the magnetic reconfiguration method of an assembly. By performing the cycle in the (B_x, B_y) space (described in Fig. 3 and 4), the different transitions shown in Fig. 2 can also be obtained in simulations. An example of transition is shown in Fig. S2 between S16b and S16d. The state of the assembly is presented at different stages of the transition in order to compare experiments and simulations. The first line of configurations correspond to experimental results and the second line correspond to simulations. The study of the potential of the structure allows us to correctly simulate the transition between 2 states. As the initial conditions are different for each simulation, it is impossible to predict which transition will be observed. Even with the same initial particles' distribution, since the model is stochastic, it may converge to different states on different runs as in experiments.

However, using this potential calculation is not optimal to model the controlled growth method. Indeed, this model does not correctly predict the dynamics of a new particle approaching an existing crystal. We will therefore model this technique by looking at the forces rather than the potential in the following section.

Dynamic modeling

Although the previously described static model is reliable to find energy minima, using the steepest descent algorithm, it may not always produce physical trajectories. In particular, when a set of particles is at equilibrium, but one or more other particles are placed far away from the set, overall, the system is far from equilibrium. In that case, the steepest descent algorithm may produce trajectories that distort the subset of particles while trying to reach a global equilibrium. This situation does not arise with the magnetic reconfiguration method, since we start from a global equilibrium configuration, and then modify the in-plane field to trigger the reorganizations. But in the controlled

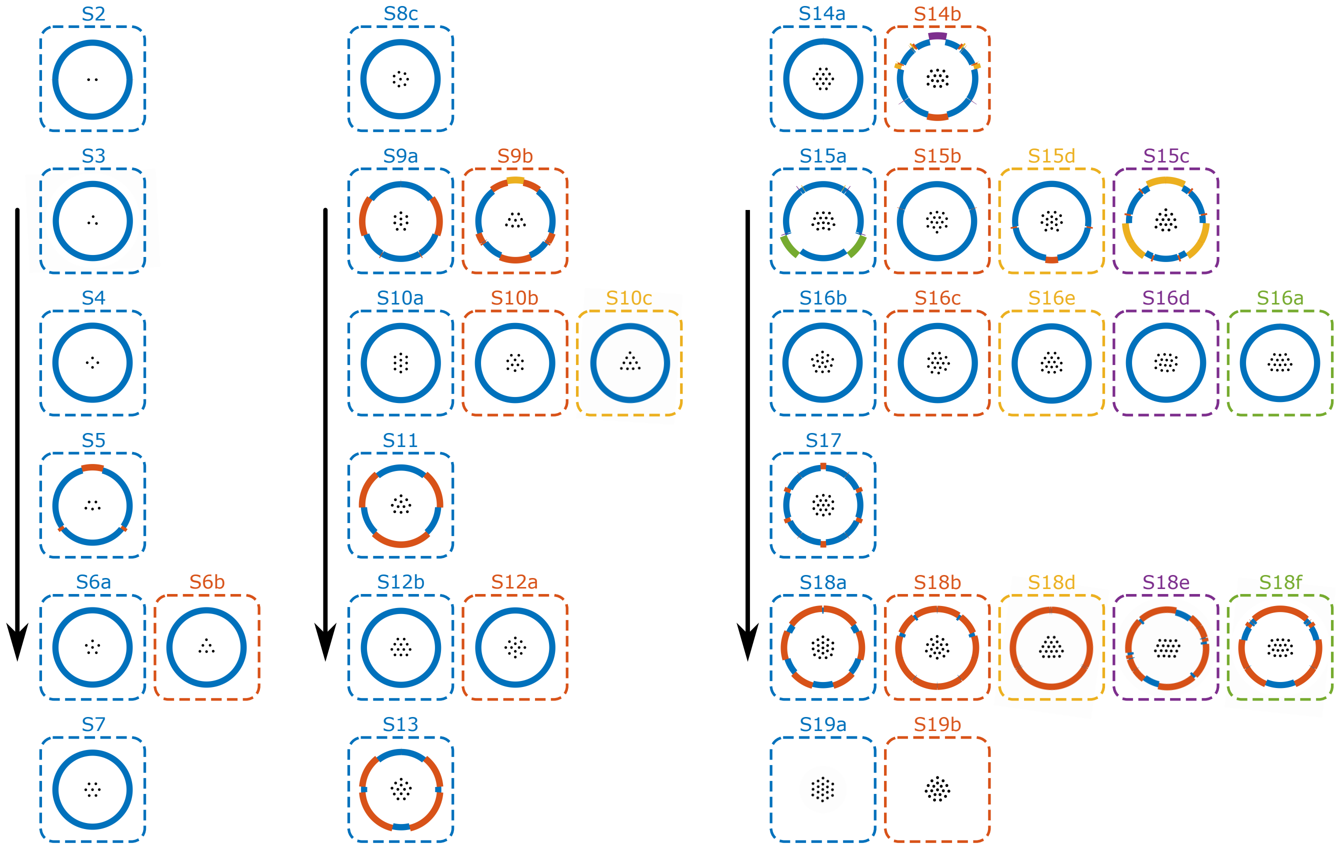

Figure S3. Summary of the dynamic simulations results of the transitions from an N crystal to an N+1 crystal for an external magnetic field $B_z = 5\,\mathrm{mT}$. The added particle is placed at five capillary lengths of the initial assembly and at different angles. The color of each assembly is represented by the dashed square around it. The first state of each row (in a blue square) is the global energy minimum, the subsequent states are in increasing order of energy. The color ring around each assembly represents the possible transitions to assemblies of the following row if a particle is added in that direction. For example, adding a particle "south" of S5 (blue color ring region) renders S6a (blue dashed square), while adding it "north" (orange color ring region), renders S6b (orange dashed square).

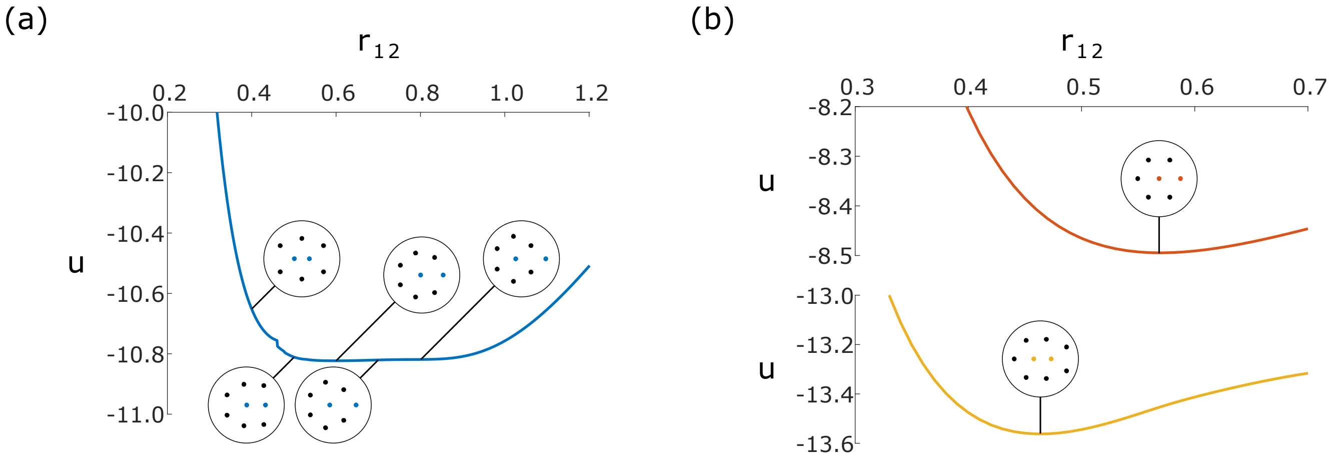

Figure S4. Dynamic simulations results for an N particles assembly for an external magnetic field $B_z = 5\,\mathrm{mT}$. In this case, the interdistance of the two colored particles was imposed, and the rest were let free to rearrange around them. We show the assembly energy u as a function of the imposed interdistance r_{12}. (a) For an 8 particles assembly, we see that the energy minimum is not well-defined, allowing the coexistence of several states. (b) In contrast, for a 7 and 9 particles assembly, we observe a well defined minimum.

growth method, this is always the case since we start from an equilibrium configuration and we add one particle far away. Therefore, for this case we developed a deterministic dynamic model that consists in computing the forces acting on each particle.

First, the dimensionless pairwise interaction force is computed as the gradient of the pairwise dimensionless potential (Eq.(1)),

$$\vec{f}_{ij} = \left(K_1\left(x_{ij}\right) - 3\frac{\mathrm{Mc}}{x_{ij}^4} \right) \hat{r}_{ij}, \tag{S2}$$

where $\vec{f}_{ij}$ is the dimensionless force exerted by the particle j on the particle i, and $\hat{r}_{ij}$ is the unitary vector pointing from particle i to particle j. Second, the total force acting on each particle is computed as

$$\vec{f}_i = \sum_{\forall j \neq i} \vec{f}_{ij}. \tag{S3}$$

Then, neglecting the inertia of the particle (viscosity dominated scenario), the velocity of the particle is approximated as proportional to the force ($\vec{v}_i \propto \vec{f}_i$) as predicted by the Stokes law for low Reynolds flows. Finally, it suffices to numerically integrate each particle position as

$$\vec{x}_{i_{n+1}} = \vec{x}_{i_n} + \alpha \vec{f}_i, \tag{S4}$$

where n is the actual time step and α acts as an integration time step. We found that, for $\alpha < 10^{-2}$, the system converges, but we chose $\alpha = 10^{-3}$ for robustness.

This dynamic model was used to predict the evolution of the system when a new particle is added to an existing crystal. It can predict the resulting state as a function of the new particle position with respect to the original crystal. Indeed, it produced the results shown in Fig. 6, where a S5 crystal transitioned into a S6a or S6b crystal depending on the position of the new particle. Similar results were obtained for crystals made of 3 to 19 particles and are summarized in Fig. S3. The first discrepancy we identify between simulation and experimental results (Fig. 2) is that, for an eight particles crystal, only one stable state (S8c) is predicted. This can be explained by the fact that the energy landscape around equilibrium is quite flat, as suggested by the results shown in Fig. S4. In that case, any variation in the model (e.g., short distance capillary interactions, small inhomogeneities between

the particles) or not considered effect (e.g., immersion depth variation) can make the system fall into one state or another. A similar scenario is suspected to happen for S18b and S18c, where only S18b is predicted stable by the simulation. Additionally, although five possible states are predicted for an 18 particles assembly, only S18a and S18b are obtainable from S17 which agrees with experimental observations. On the other hand, although the numerical simulations distinguish S15b from S15d, experimentally the were considered the same since the central particles have the same symmetry. Finally, some transitions are predicted to happen only when placing the new particle in a precise location (e.g., from S15a to S16d), which is not realizable experimentally. Besides the previously mentioned cases, all the predicted transitions were observed experimentally.

D. States occurrence frequency

Although in the paper we focused on the controlled transitions between metastable states, we can also study the frequency of appearance of each state from an initially random distribution of particles. Such study would be difficult to perform experimentally, since placing the particles sequentially on the surface would not give place to a random distribution; the particles will tend to agglomerate while being placed. On the other hand, this can be easily implemented in simulations since all particles can be randomly placed simultaneously.

We have performed numerical simulations, using the dynamic model described in the Supplementary Materials, starting from an initially random distribution of particles. The results are shown in Fig. S5, where we showed the different possible metastable states and their frequency of occurrence. It can be noticed that the stable/ground state (minimal energy) is not always the most common one. Interestingly enough, the rate of appearance evolves differently for different number of particles. Indeed, for N = 6, 9, 10, 16 and 18, the stable states is the most common one, while for N = 12, 14, 15 and 19, one of the metastable state is more common.

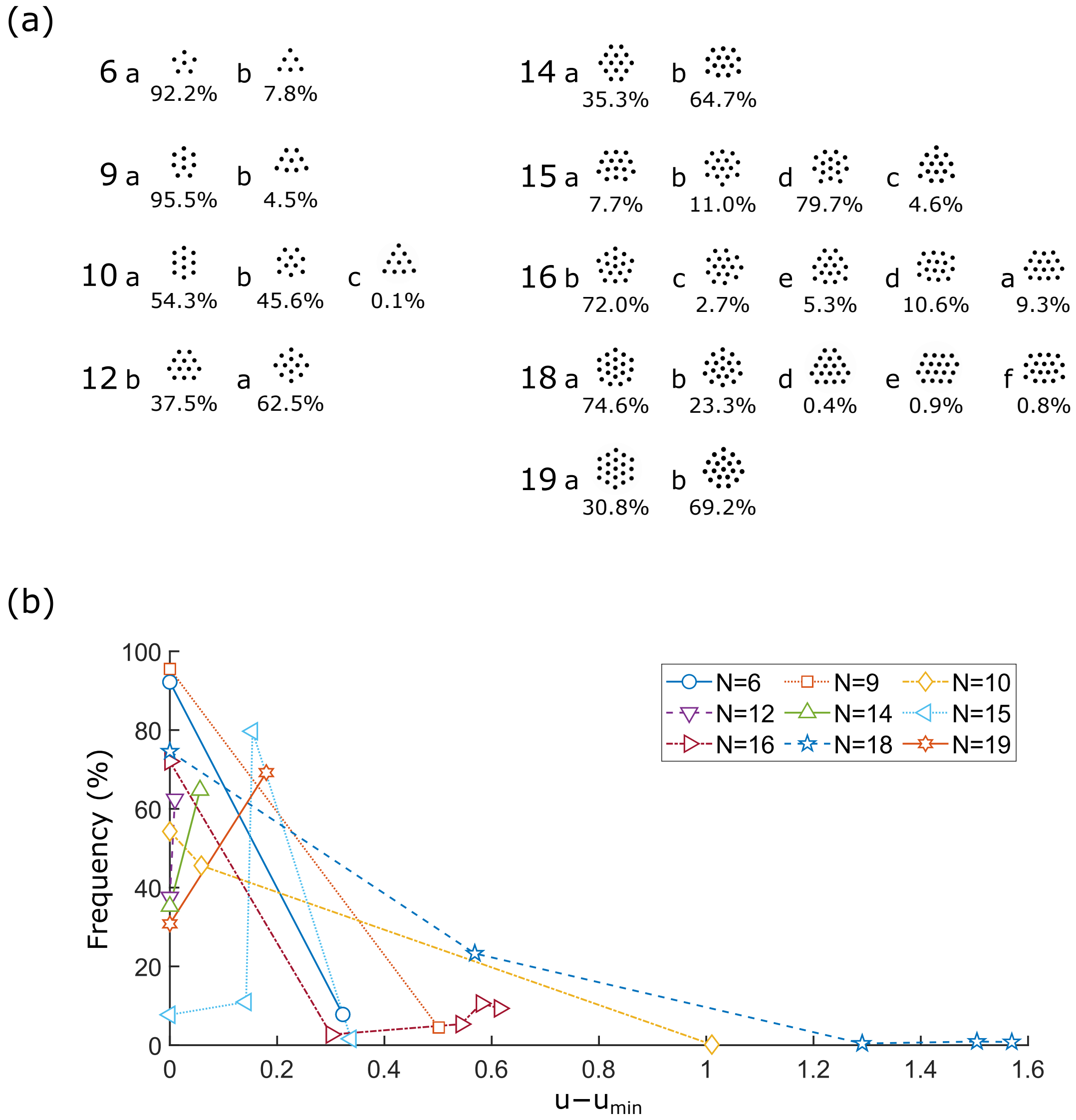

Figure S5. Metastable configurations found through numerical simulations (dynamic modeling) for different number of particles. We performed 5000 simulations for each number of particles (except for $N = 10$, for which we performed 25000), starting with a random distribution of particles in a $(8 \times 8)\,\lambda$ workspace. (a) Found configurations and their appearance frequency. The states are organized from left to right in an increasing energy order. (b) Appearance frequency as a function of the normalized energy of the configuration (with respect to the energy minimum).

References

1. Vella, D. Floating versus sinking. *Annu. Rev. Fluid Mech.* **47**, 115–135 (2015).
2. Vella, D. & Mahadevan, L. The "cheerios effect". *Am. J. Phys.* **73**, 817–825 (2005).
3. Vandewalle, N. *et al.* Symmetry breaking in a few-body system with magnetocapillary interactions. *Phys. Rev. E* **85**, 041402 (2012).
4. Grosjean, G., Hubert, M., Lagubeau, G. & Vandewalle, N. Realization of the najafi-golestanian microswimmer. *Phys. Rev. E* **94**, 021101 (2016).

5. Collard, Y., Grosjean, G. & Vandewalle, N. Magnetically powered metachronal waves induce locomotion in self-assemblies. *Commun. Phys.* **3**, 1–10 (2020).

6. Grosjean, G. *et al.* Remote control of self-assembled microswimmers. *Sci. Reports* **5**, 1–8 (2015).

7. Vandewalle, N., Obara, N. & Lumay, G. Mesoscale structures from magnetocapillary self-assembly. *The Eur. Phys. J. E* **36**, 1–6 (2013).

8. Hubert, M. *et al.* Scallop theorem and swimming at the mesoscale. *Phys. Rev. Lett.* **126**, 224501 (2021).

9. Scriven, L. E. & Sternling, C. V. The Marangoni effects. *Nature* **187**, 186–188, DOI: 10.1038/187186a0 (1960).

10. Piñan Basualdo, F. N. *et al.* Effect of insoluble surfactants on a thermocapillary flow. *Phys. Fluids* **33**, 072106 (2021).

11. Piñan Basualdo, F. N., Bolopion, A., Gauthier, M. & Lambert, P. A microrobotic platform actuated by thermocapillary flows for manipulation at the air-water interface. *Sci. Robotics* **6**, eabd3557 (2021).

12. Chraïbi, H. & Delville, J.-P. Thermocapillary flows and interface deformations produced by localized laser heating in confined environment. *Phys. Fluids* **24**, 032102 (2012).
